# Refractory hypokalemia associated with levetiracetam: a case report

**DOI:** 10.11604/pamj.2026.53.87.51338

**Published:** 2026-02-17

**Authors:** Mohamed Ksentini, Rim Atheymen, Lobna Ben Mahmoud, Nour El Houda Ben Ali, Ahmed Hakim, Khaled Zghal, Brahim Kammoun

**Affiliations:** 1 Regional Pharmacovigilance Service in Sfax, Sfax, Tunisia,; 2 Department of Pharmacology of Sfax, Faculty of Medicine of Sfax, Sfax University, Sfax, Tunisia,; 3 Laboratory of Research of Pharmacology and Toxicology (LR19ES10), University of Sfax, Sfax, Tunisia,; 4 Department of Neurosurgery, Habib Bourguiba Hospital of Sfax, University of Sfax, Sfax, Tunisia

**Keywords:** Levetiracetam, hypokalemia, drug safety, pharmacovigilance, case report

## Abstract

Levetiracetam (LEV) is frequently prioritized in epilepsy management due to its minimal drug-drug interactions and perceived metabolic neutrality. However, its potential involvement in severe electrolyte disturbances remains scarcely documented. We describe a 36-year-old male admitted for status epilepticus and initiated on a dose-escalated LEV regimen (2000 mg/day). The clinical course was complicated by the development of severe, recalcitrant hypokalemia (nadir 2.8 mmol/L). This electrolyte depletion proved remarkably refractory to intensive parenteral and enteral potassium chloride supplementation. A systematic diagnostic workup successfully excluded extrarenal losses, primary hyperaldosteronism, and renal tubular acidosis. Given the specific temporal alignment with drug intensification, LEV was substituted with phenobarbital. This intervention led to a rapid and sustained restoration of potassium homeostasis within seven days post-withdrawal. Causality was evaluated as highly probable (I5; C2S3). This case, corroborated by recent large-scale epidemiological findings, highlights LEV as a potential trigger for refractory potassium depletion in the adult population. Unlike other antiepileptics, LEV lacks carbonic anhydrase inhibition, suggesting alternative mechanisms such as iatrogenic transcellular shifts or interference with renal ion conductance. We emphasize the necessity of rigorous metabolic monitoring during LEV dose escalation and advocate for an update to official safety labeling to include this metabolic risk, ensuring earlier clinical detection and improved patient safety.

## Introduction

Levetiracetam (LEV) has established itself as a cornerstone in the modern management of epilepsy, primarily due to its pharmacokinetic predictability and perceived safety profile [1,2]. Its distinct mechanism of action, characterized by a lack of hepatic metabolism and minimal plasma protein binding, has made it a preferred choice to avoid complex drug-drug interactions. However, as its clinical use expands globally, rare but clinically significant idiosyncratic reactions are emerging that challenge its reputation as a metabolically neutral agent [3,4]. Among these, severe electrolyte imbalances-specifically hypokalemia-remain an under-recognized and potentially life-threatening complication that is seldom documented in current literature. While LEV is traditionally considered safe for the internal milieu, we describe a case of severe, recalcitrant hypokalemia triggered by LEV dose escalation, highlighting a critical metabolic risk that warrants closer pharmacological scrutiny. The causality was evaluated using the Miremont-Salamé *et al*. updated French imputability criteria [5].

## Patient and observation

**Patient information and timeline of current episode:** a 36-year-old male, with a surgical history of ventriculoperitoneal shunting for childhood hydrocephalus, was admitted to the medical intensive care unit in October 2024 following an episode of status epilepticus. Therapeutic stabilization necessitated a multimodal antiepileptic strategy, including sodium valproate and a dose-escalated regimen of levetiracetam (LEV) at 2000 mg/day.

**Clinical findings:** the clinical course was further complicated by infectious meningoencephalitis, managed with a combination of aciclovir, imipenem, and colimycin. Despite this complex pharmacological environment, laboratory monitoring on November 15 identified a sudden and severe drop in serum potassium, reaching a critical nadir of 2.8 mmol/L. This hypokalemia proved remarkably recalcitrant; intensive parenteral and enteral potassium chloride (KCl) supplementation failed to sustain physiological levels.

**Diagnostic assessment:** a systematic diagnostic workup was initiated, which successfully excluded extrarenal losses, primary hyperaldosteronism, and renal tubular acidosis. Notably, the other co-prescribed molecules were ruled out as primary triggers given the specific temporal alignment with LEV intensification.

**Therapeutic interventions:** on November 27, following a multidisciplinary review, LEV was electively discontinued and substituted with phenobarbital.

**Follow-up and outcome of interventions:** this intervention led to an immediate and sustained restoration of potassium homeostasis within seven days, without further requirement for exogenous supplementation. The evolution of serum potassium levels throughout the episode is summarized in Table 1. The causality was rigorously evaluated using the Miremont-Salamé *et al*. updated French imputability criteria, yielding a high-probability score of I5 (C2S3). The case was subsequently validated and registered at the Regional Pharmacovigilance Center of Sfax (Ref: 900/2024).

**Table 1:** evolution of serum potassium levels before and after the onset of hypokalemia under levetiracetam

Date	Serum Potassium (mmol/L)	Potassium Supplementation	Levetiracetam intake
August 2024	3.9	None	Yes (500 mg/day)
14/11/2024	4.1	None	Yes (500 mg/day)
15/11/2024	3.1	3 ampoules KCl	Yes (500 mg/day)
21/11/2024	2.3	3 ampoules KCl	Yes (500 mg/day)
25/11/2024	2.7	3 ampoules KCl	Yes (500 mg/day)
27/11/2024	2.7	None	Discontinued
04/12/2024	4.1	None	No
20/12/2024	4.0	None	No

**Patient perspective:** the patient reported having been unaware of the metabolic imbalance during the acute phase due to his neurological condition. However, upon recovery, he expressed relief at the resolution of the biological disturbances and the stabilization of his seizures following the adjustment of his medication. He emphasized the importance of close monitoring, which allowed the medical team to identify this unexpected side effect.

**Informed consent:** the case was reported to the regional pharmacovigilance service as part of routine pharmacovigilance. The case report is presented anonymously, and all identifying information has been removed to protect patient confidentiality.

## Discussion

While levetiracetam has long been celebrated for its pharmacological neutrality, our clinical observation adds to a growing body of evidence suggesting that its metabolic impact may be more significant than previously recognized [6,7]. To date, LEV-induced hypokalemia remains a scarcely characterized adverse drug reaction (ADR), with fewer than five documented cases in the literature. However, this clinical rarity has recently been challenged by the epidemiological weight of the 2024 Almadani *et al*. cohort study. By identifying a two-fold increased risk of hypokalemia compared to carbamazepine, with a reported incidence of nearly 3%, their findings provide a necessary statistical foundation for what was previously considered anecdotal evidence [8].

From a mechanistic perspective, the molecular underpinnings of this disturbance remain to be fully elucidated. Unlike topiramate or zonisamide, LEV does not exhibit significant carbonic anhydrase inhibition, which necessitates the exploration of alternative pathways [9]. The 'recalcitrant' nature of the potassium depletion observed in our patient, which persisted despite aggressive and sustained supplementation, strongly suggests a profound disruption of homeostatic handling. This could stem from an iatrogenic transcellular shift or, more likely, a direct interference with renal tubular ion conductance. Such a phenomenon might act as a 'stress test' for latent tubular dysfunction, revealing subclinical vulnerabilities in the mature kidney.

Furthermore, the apparent pediatric resilience to these electrolyte perturbations documented in prospective trials-contrasts sharply with the emerging susceptibility in the adult population [10]. This age-dependent dichotomy suggests that the molecular targets of LEV in the renal tubule or the systemic ion-exchange mechanisms may undergo maturational changes, rendering adults more vulnerable to this specific iatrogenic risk. Our case underscores the imperative for clinicians to move beyond the 'set-and-forget' mentality regarding LEV's safety and to maintain rigorous metabolic vigilance in adult patients.

## Conclusion

Ultimately, this clinical observation serves as a pharmacological alert, highlighting the imperative for systematic electrolyte surveillance in adult patients, particularly during levetiracetam dose intensification. When clinicians encounter recalcitrant hypokalemia of cryptic origin, LEV-induced depletion should be integrated into the differential diagnosis as a plausible iatrogenic entity. Given the expanding global prescription of this antiepileptic agent, we advocate for a formal update of the Summary of Product Characteristics (SmPC) to include this metabolic risk. Such regulatory recognition is essential to facilitate earlier clinical detection, mitigate potentially severe cardiovascular or muscular complications, and ultimately refine the safety framework of modern antiepileptic therapy.
